# A New Differential Diagnosis of Dysphagia: A Case Report of Lymphocytic Esophagitis

**DOI:** 10.7759/cureus.13010

**Published:** 2021-01-30

**Authors:** Fernando Resende, António Ferrão, Renato Melo, Elisabete Barbosa, Adelino Barbosa

**Affiliations:** 1 General Surgery, Centro Hospitalar e Universitário de São João, Porto, PRT

**Keywords:** deglutition disorders, differential diagnosis, esophagitis, lymphocytes

## Abstract

Lymphocytic esophagitis is a rare but increasingly recognized cause of chronic esophagitis. The pathogenesis, prognosis, and treatment are undefined. We report the diagnostic workup of an unusual cause of dysphagia. We present a case report of a 71-year-old female who presented with dysphagia for solid foods. The endoscopic appearance showed stenosis at the cricopharyngeus and trachealization of the proximal esophagus. Biopsies were taken to exclude eosinophilic esophagitis. The pathology showed lymphocytic infiltrate with peripapillary distribution with no granulocytes and spongiosis suggestive of lymphocytic esophagitis. Esomeprazol was started with symptomatic improvement. The symptoms and endoscopic appearance of lymphocytic esophagitis may be indistinguishable from other forms of chronic esophagitis. A high index of suspicion and mucosal sampling are essential to establish the diagnosis. Lymphocytic esophagitis seems to be a chronic and benign form of esophagitis. It should be included in the differential diagnosis of dysphagia. Further research and case reporting are essential to better define its pathogenesis, prognosis, and treatment.

## Introduction

Lymphocytic esophagitis is a rare form of chronic esophagitis. It was first described in 2006 by Rubio et al. [1]. Lymphocytic esophagitis was found in 0.09% of esophageal biopsies obtained by endoscopy in a study of 129,252 adult patients [2]. The characteristic histologic pattern consists of a lymphocytic intraepithelial infiltration with a peripapillary distribution associated with no or few granulocytes and spongiosis [3].

This increasingly recognized condition should be part of the differential diagnosis of dysphagia. Its etiology and clinical implications are yet to be fully elucidated; as such, reporting these patients is essential to elucidate the natural history and prognosis of this new entity.

We describe a case of a female patient presenting to a tertiary and academic center complaining of dysphagia. This case report has been reported in line with the surgical case report (SCARE) criteria [4].

## Case presentation

A 71-year-old Caucasian female patient presented to the general surgeon appointment complaining of dysphagia, after initial evaluation in the general practice clinic. She is retired but used to work in a textile manufacturer. The patient had a previous medical history of arterial hypertension, osteoarthritis, and incomplete CREST (calcinosis, Raynaud's phenomenon, esophageal dysmotility, sclerodactyly, and telangiectasia) syndrome and was medicated with glucosamine, calcium supplements, and amlodipine (10 mg, once daily). She had no known medication allergies and no history of tobacco or alcohol consumption; the patient also denied chronic use of nonsteroidal anti-inflammatory drugs. The familial medical history was unremarkable.

The dysphagia was present for two years for solid foods only. The patient avoided this kind of food but otherwise had no other restrictions on her diet. She also complained of sporadic dyspeptic symptoms. The patient had no other symptoms namely nausea, vomiting, sialorrhea, anorexia, weight loss, or abdominal pain. The physical exam was unremarkable.

The diagnostic workup started in the general practice clinic with an upper endoscopic study and esophageal manometry. The endoscopy was incomplete due to stenosis at the cricopharyngeus not allowing passage of the standard endoscope; no biopsies were taken. The esophageal manometry showed dysmotility of the distal two-thirds of the esophagus. The patient was then referred to a general surgery appointment in our institution.

A second endoscopic study was performed to achieve mucosal sampling and try to surpass the stenosis. This was possible with a 55 mm scope and the mucosa of the proximal esophagus showed trachealization with no other relevant alterations. The stomach and the duodenum were normal. Biopsies were taken from the stenosis and the adjacent mucosa of the esophagus (both proximally and distally).

**Figure 1:**
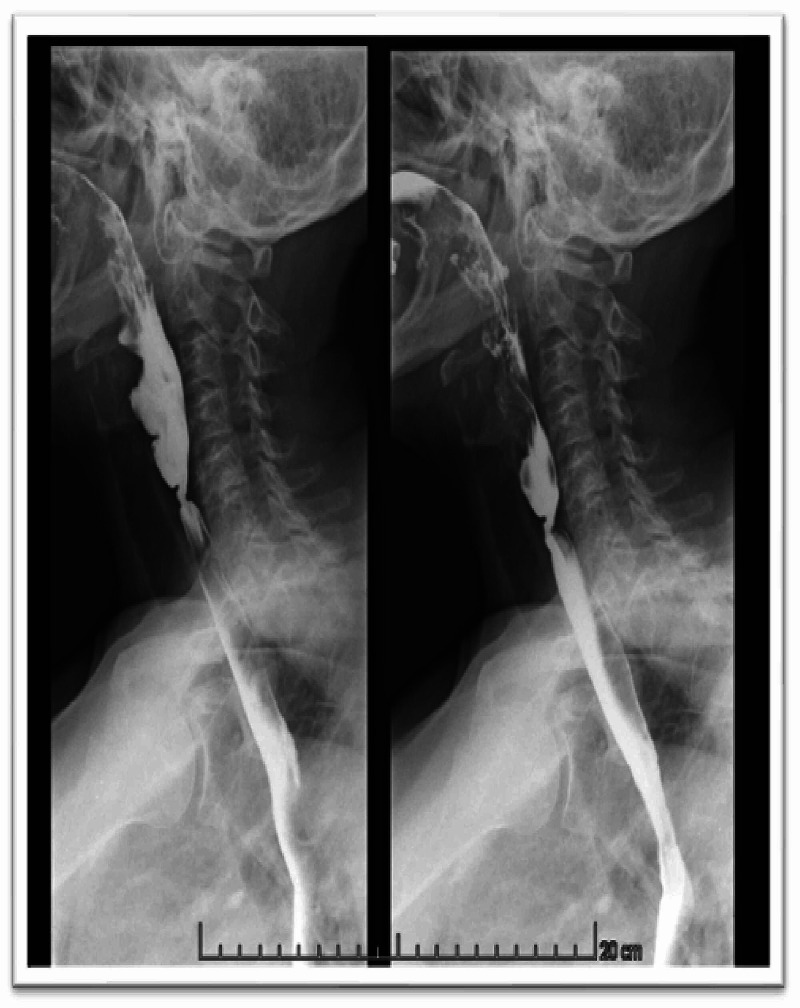
Barium swallow study The barium swallow study did not reveal any esophageal diverticula, stenoses or hiatus hernia or signs of esophageal motility disorders.

A barium swallow study and a second manometry were also performed. The former did not reveal any esophageal diverticula, stenoses or hiatus hernia and both showed no signs of esophageal motility disorders (Figure 1). There was no anemia, abnormal leucocyte count or elevated C-reactive protein in the blood analysis.

**Figure 2:**
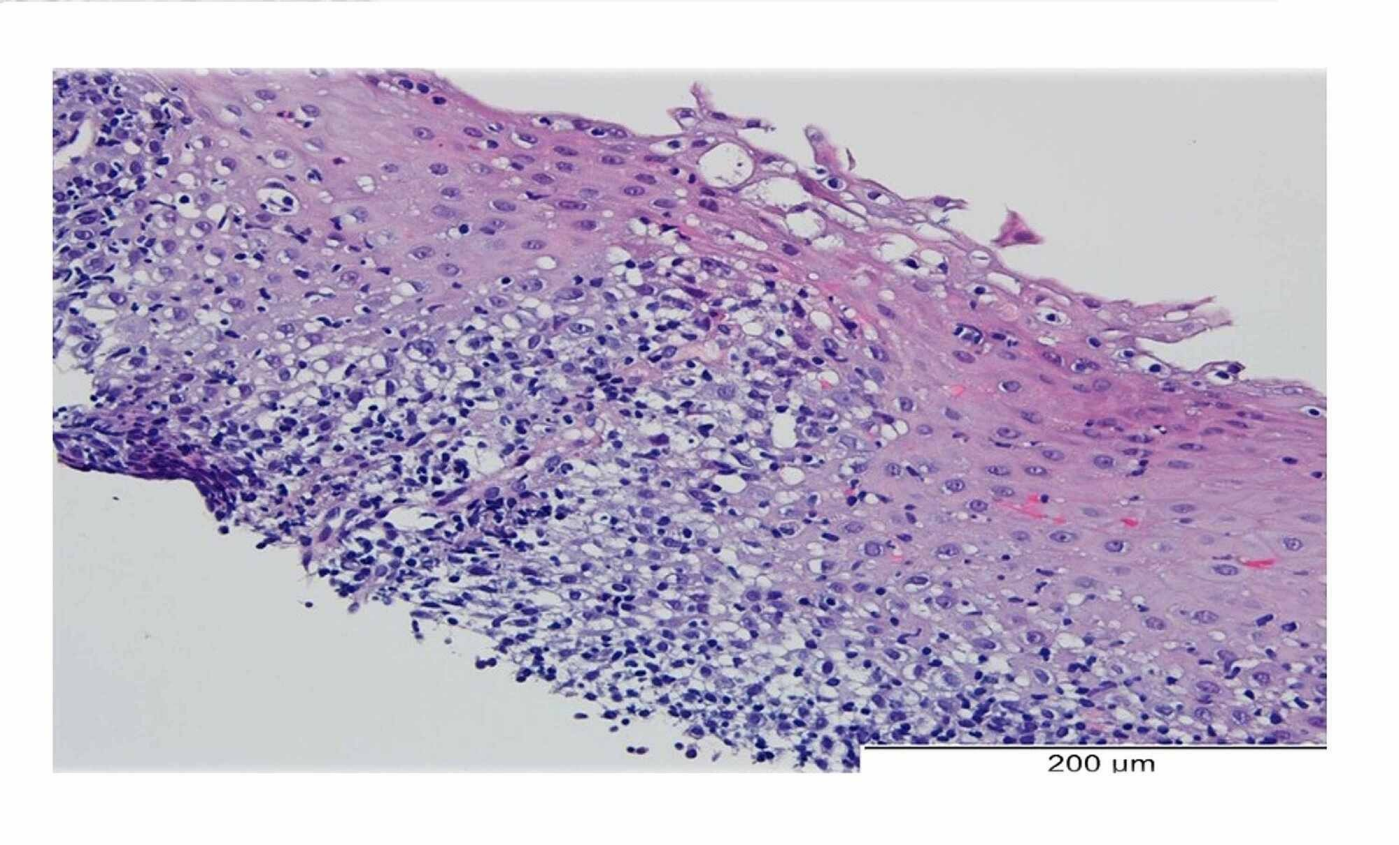
Esophageal biopsy The esophageal biopsies revealed mucosa with a stratified squamous epithelium with lymphocytic infiltrate in a peri-papillary distribution and spongiosis. No granulocytes were observed.

The biopsies revealed mucosa with a stratified squamous epithelium with lymphocytic infiltrate in a peri-papillary distribution and spongiosis. No granulocytes, namely eosinophils, were observed (Figure 2). There were no signs of malignancy or dysplastic alterations. The pathologic study concluded that there was chronic inflammation of the mucosa compatible with lymphocytic esophagitis.

The patient was started on esomeprazole 40 mg, once daily. Four months later, she referred symptom improvement being able to swallow solid foods, complaining only of sporadic heartburn. Nowadays she maintains acid-suppressing therapy and follow-up by a general surgeon. She has an upper endoscopic procedure and repeated mucosal sampling scheduled at one year after the initial evaluation.

## Discussion

Lymphocytic esophagitis is a rare but increasingly recognized cause of dysphagia and, as such, should be part of the diagnostic algorithm of these patients. The diagnosis requires a high index of suspicion since its symptoms are non-specific and there is no age or gender predilection. Patients often complain of heartburn, dyspepsia, abdominal pain, nausea, and vomiting but dysphagia seems to be present in approximately 57% of the patients. Food impaction can also occur [5].

Endoscopy and mucosal sampling are essential to the diagnosis. Nonetheless even the endoscopic appearance can vary widely from “normal” appearing mucosa (33% of the patients) to narrowing, esophageal rings, linear furrows, and trachealization [6]. Notably, approximately 40% of the patients may exhibit an endoscopic appearance indistinguishable from eosinophilic esophagitis. In this scenario, mucosal sampling assumes a pivotal role, showing the characteristic peripapillary infiltration of lymphocytes and absence of granulocytes, namely eosinophils, in association with spongiosis that makes the diagnosis of lymphocytic esophagitis.

There are few reports on the efficacy and availability of the different treatment options for lymphocytic esophagitis. In fact, most of the treatments applied were extrapolated from other forms of chronic esophagitis. Acid suppressing therapy with proton-pump inhibitors (PPI) is one of the most commonly applied strategies and seems to confer symptom improvement but the actual mechanism of actions is unknown. Some authors attribute this effect to the anti-inflammatory properties of PPIs while others advocate the improvement to amelioration of superimposed gastric reflux symptoms. Endoscopic dilation is also an important consideration especially in the setting of dysphagia and esophageal stenosis. This technique seems to ameliorate symptoms and it could be repeated, as long as both the patient and the practitioner are fully aware of its benefits and possible iatrogenesis [7]. There is anecdotal report that topical corticosteroid therapy and dietary changes could have some effect on symptoms but there is a lack of evidence supporting these measures.

Due to the rarity of this condition, its natural history and pathogenesis are yet to be fully elucidated. It is believed that this a chronic and benign form of esophagitis although there are some cases of serious morbidity, namely esophageal perforation [8]. Some authors hypothesize that lymphocytic esophagitis may be associated with alcohol or tobacco use but the only association that achieved statistical significance was Crohn's disease, namely in the pediatric population [9,10]. However, the same association was not found for the adult population. It is also worth mentioning that it seems to be a subset of lymphocytic esophagitis associated with esophageal motility disorders. In fact, Xue et al. [11] demonstrated that cluster of differentiation (CD)4+ T-cells are predominant in patients with both lymphocytic esophagitis and esophageal dysmotility while in those with normal esophageal motility the lymphocytic infiltrate consisted mainly of CD8+ T-cells. This observation warrants further evidence being one of the future research topics on lymphocytic esophagitis.

## Conclusions

Lymphocytic esophagitis is a rare but increasingly recognized condition. Since its symptoms and endoscopic appearance are non specific, a high degree of suspicion, mucosal sampling, and histopathological analysis are essential to the diagnosis. Clinicians should include lymphocytic esophagitis in the differential diagnosis of chronic esophagitis and dysphagia. Further research and report of known cases will help to elucidate the natural history, prognosis, and treatment of this novel entity.
